# Non-compliance with a nurse’s advice to visit the primary care provider: an exploratory secondary analysis of the TRIAGE-trial

**DOI:** 10.1186/s12913-022-07904-8

**Published:** 2022-04-08

**Authors:** Ines Homburg, Stefan Morreel, Veronique Verhoeven, Koenraad G. Monsieurs, Jasmine Meysman, Hilde Philips, Diana De Graeve

**Affiliations:** 1 Department of Economics, University of Antwerp, Antwerp, Belgium; 2 Department of Family and Population Health, University of Antwerp, Antwerp, Belgium; 3 Department ASTARC, Antwerp University Hospital, University of Antwerp, Antwerp, Belgium

**Keywords:** After-hours care, Emergency department, Primary care, Triage, Non-compliance, General practitioners cooperative, Costs

## Abstract

**Background:**

During the cluster randomised TRIAGE-trial, a nurse advised 13% of low-risk patients presenting at an emergency department in Belgium to visit the adjacent general practitioner cooperative. Patients had the right to refuse this advice. This exploratory study examines the characteristics of refusers by uncovering the determinants of non-compliance and its impact on costs, as charged on the patient’s invoice.

**Methods:**

Bivariate analyses with logistic regressions and T-tests were used to test the differences in patient characteristics, patient status, timing characteristics, and costs between refusers and non-refusers. A chi-square automatic interaction detection analysis was used to find the predictors of non-compliance.

**Results:**

23.50% of the patients refused the advice to visit the general practitioner cooperative. This proportion was mainly influenced by the nurse on duty (non-compliance rates per nurse ranging from 2.9% to 52.8%) and the patients’ socio-economic status (receiving increased reimbursement versus not OR 1.37, 95%CI: 0.96 to 1.95). Additionally, non-compliance was associated (at the 0.10 significance level) with being male, not living nearby and certain reasons for encounter. Fewer patients refused when the nurse perceived crowding level as quiet relative to normal, and more patients refused during the evening. The mean cost was significantly higher for patients who refused, which was a result of more extensive examination and higher out-of-pocket expenses at the ED.

**Conclusions:**

The nurse providing the advice to visit the general practitioner cooperative has a central role in the likelihood of patients’ refusal. Interventions to reduce non-compliance should aim at improving nurse-patient communication. Special attention may be required when managing patients with a lower socio-economic status. The overall mean cost was higher for refusers, illustrating the importance of compliance.

**Trial registration:**

The trial was registered on registration number NCT03793972 on 04/01/2019.

## Background

Crowding of emergency departments (EDs) in hospitals is a commonly reported problem, particularly out-of-hours (OOH). Although there is no consensus on the definition of ‘appropriate’ or ‘inappropriate’ use of the ED, several studies found that many medical problems presented at the ED could be managed in a primary care setting, as they do not always require emergency care [1–4] In many European countries, OOH primary care is organised in General Practitioners Cooperatives (GPCs). These GPCs operate as walk-in centres for unplanned OOH care, thus offering an alternative for ED visits, and are staffed by the regional GPs. In Belgium, approximately 80 cooperatives have been introduced from 2003 onwards, covering about 70% of the population [5, 6] The organisation of these cooperatives improved access to OOH primary care and was associated with an increased use of primary care. However, GPCs did not necessarily lead to a decrease in the workload of EDs [7] Patients with low-risk complaints, easily treatable by a GP, continue to make emergency visits, as most patients base their decision on previous experience, ease of access, the anticipated waiting time, the relationship with their general practitioner (GP), or the perceived nature of the complaints [8, 9] These visits can be a problem because, when EDs are already crowded, they may compromise efficient use of healthcare personnel, infrastructure, and financial resources. Therefore, measures should be taken to assist the patients in choosing the recommended place of care [6] Triaging patients is one possible solution. In general, triaging is defined as sorting out and classifying patients to determine treatment priority and proper place of treatment [10] Current ED triage systems are only used to determine the urgency of emergency treatment. Our extended triage system adds assignment to the ED or GPC to this system. However, little is known about the effectiveness and safety of this system [11].

The TRIAGE-trial determined the impact of a nurse-led triage system that assigned low-risk patients from the ED to the adjacent GPC. At the time of the current study, Belgian GPCs were only open during weekends and bank holidays. A newly developed extension to the Manchester Triage System (eMTS) was used to identify patients with low urgency complaints and advise them during intervention weekends to visit the GPC. During control weekends, the advice was recorded but not communicated to patients, who therefore all remained at the ED. The study showed that during intervention weekends 838/6294 (13.3%, 95%CI: 12.5 to 14.2) of patients received the advice to visit the GPC of which 196/838 (23.4%, 95%CI: 20.6 to 26.4) refused. During control weekends, the fraction of patients assigned to the GPC was twice as high, indicating nurses may find it easier to give theoretical advice rather than discuss it with the patient. During the entire trial, 2.4% (95%CI: 1.7 to 3.4) of the patients assigned to the GPC were admitted to the hospital. Unfortunately, one patient diverted to the GPC deceased due to a ruptured abdominal aneurysm. Nevertheless, the trial showed that a sustainable safe relocation of non-urgent ED patients to primary care is possible using the eMTS. The authors highlight the need for further research and multicentre studies to improve the tool and guarantee safe relocation [12].

The conclusion that such relocation is feasible is confirmed by smaller, non-randomised, studies as well [13–16] However, the role of patients who refuse the advice to visit the GPC is often omitted. Such non-compliance undermines the effectiveness of the system, yet not much is known on this subject. The determinants of non-compliance to general medical treatment have been researched, but no theoretical framework exists that adequately predicts the behaviour. For instance, authors find contradictory results on the role of patient’s sex and age [17] Most studies find that socio-economic characteristics, such as unemployment or low-income contribute to non-compliance [18, 19], although educational level does not seem to be a predictor [20] Overall, high rates of non-compliance have been reported in multiple settings and across many socio-demographic groups. Estimates of its overall rate range from 30 to 50% and above [21].

The TRIAGE-trial offers a unique opportunity to examine non-compliance further. This article examines the patients who were assigned to the GPC during the nurse-led triage but refused the advice and were treated at the ED. This article investigates how large the proportion of refusers was, what the determinants were, and compares the costs of the provided medical services between compliers and non-compliers, as captured on the invoice that patients received from the ED or GPC. Although an exploratory analysis does not allow to determine causality, the results may give valuable insights into the refusal of medical advice and its financial consequences. They may allow us to formulate suggestions for interventions aimed at reducing the refusal rate.

## Methodology

### The TRIAGE-trial

The TRIAGE-trial was set up to determine the effectiveness and safety of a nurse-led triage system that assigns low-risk patients from an ED to the GP. A single-centre cluster randomised trial was performed with weekends and bank holidays (hereinafter called weekends) serving as units of randomisation and patients as units of analysis. The trial ran from 01/03/2019 to 30/12/2019. During intervention weekends, patients were assigned to a particular care setting but they had the possibility to refuse: low-risk patients were considered as candidates for primary care and were assigned to the GPC, while patients in need of more urgent or advanced care were assigned to the ED. Control weekends are not of interest in this study, as the advice was not communicated to patients and they all remained at the ED. The trial was executed in the ED of the Belgian general hospital ‘AZ Monica’ and the adjacent GPC ‘Antwerpen Oost’. The surrounding area has citizens from a variety of ethnicities and consists of both middle income and socially deprived neighbourhoods. The Belgian healthcare system is mainly organised as a fee-for-service system and is characterised by free choice and open access for patients to all medical services.

The triaging of patients was done using a locally developed extension to the MTS (eMTS). The eMTS contains the entire MTS version 3.6, one of the main triage systems used worldwide [22] The system is a tool for prioritisation in the ED, but previous studies have also used it to relocate patients. They have illustrated that the system presents an acceptable validity [13, 15, 16] The MTS is a five-level triage system and consists of 53 presentational flowcharts. Each flowchart consists of discriminators, eventually leading to an urgency category ranging from level one (immediate care necessary) to level five (non-urgent). In the adapted version, 44 flowcharts were extended with GP risk discriminators whenever the urgency category was four or five. If such discriminator was present, patients were assigned to the ED [12].

### Outcome measures

This study is a secondary analysis of the TRIAGE-trial. The predefined primary outcome is the proportion of patients that were assigned to the GPC but refused. They were treated at the ED, despite the advice to go to the GPC. The secondary outcomes of this article (not predefined) are the determinants of non-compliance and the impact on the costs.

### Data collection

The following patient characteristics were collected and used in this study: age; sex; patient lives nearby (within the four communities covered by the GPC); and socio-economic status (whether patients receive an increased reimbursement or not, which is predominantly determined by an upper bound on household income). Information on the patients’ race, education, primary language, or previous experience with the ED/GPC was unavailable. The eMTS flowchart (53 flowcharts combined into 15 categories), urgency level, type of admission to the ED (walk-in or ambulance) offered information on the patients’ presentation. Other confounders, such as their baseline health or patient distress were, however, not collected. The time period (day, evening, or night), subjective crowding at the ED (quiet, normal, or busy), and anonymous ID of the triaging nurse were also registered. All 22 nurses who performed a triage were numbered. The data from the ED and GPC were linked through their pseudonymised national insurance number using iCAREdata, which is a database for medical records during OOH care [23, 24].

After the trial, the patient-level costs of treatment at the ED and GPC were received from the billing department of AZ Monica and the GPC respectively. Both settings make use of a fee-for-service system. The data consisted of the (pseudo)nomenclature codes of all medical services provided to the patients, as captured on the invoice. The codes were grouped to reflect different cost categories: consultation fees, medical imaging, clinical biology, technical procedures, medication, hospital lump sums, and non-refundable items. Data on medical imaging or clinical laboratory tests ordered by the GP were not available at the patient level. The category non-refundable items consists of various articles at the request of the patient (e.g., a toothbrush) or necessary for their medical care (e.g., crutches). Medication costs only include medicines given to the patient during a consultation and not the prescriptions given to them. The various cost categories (except consultation fees) give insight into the treatment people received, as prices for medical services are similar for both the GPC and the ED. Consultation fees are predetermined. In Belgium, ED physicians and GPs receive different consultation fees, depending on the medical specialty of the physician and on the arrival time of the patient. For instance, under the current remuneration scheme, consultations during the night are more expensive at the GPC than at the ED, while the opposite occurs during daytime. The data also show the proportion of the invoice paid by the patient and by the national health insurance. The division is predetermined as well and depends on whether the consultation is with or without referral and on the socio-economic status of the patient. Consultations at the ED without referral require a higher share of co-payment from the patient [25] Due to anonymity, data were matched with the medical records from above on the basis of sex, birth year, postal code, and time. For nine patients (1.2%) no invoice could be matched. Ten (1.3%) patients were hospitalized. They were excluded from the financial analysis, as only their ambulant costs were available.

### Statistical methodology

The determinants of non-compliance were first considered using a bivariate analysis. The proportions of patient characteristics, patient status, eMTS components, and variables related to the time of admission were compared between refusers and non-refusers. Bivariate logistic regressions were used to calculate odds ratios. The data were analysed using JMP pro® version 14. Those variables found significant at an alpha of 0.10 were considered significant and incorporated in the multivariate analysis. A significance level of 0.10 was used since the smaller dataset and consequently larger standard errors were unlikely to produce more significant results.

A similar bivariate analysis was performed on the costs. The mean costs of compliers and non-compliers were compared using a T-test for unequal variances. A two-sided F-test for equal variance indicated this was most appropriate. A distinction was made between the fraction of the invoice paid by the national insurance and the fraction paid by the patient, as well as between the period of the day.

The multivariate analysis consisted of a chi-square automatic interaction detection (CHAID) decision tree [26, 27] This methodology is commonly used for building prediction algorithms for a target variable and can deal with large, complicated datasets in an efficient manner, without imposing a complicated parametric structure. This method classifies the population into branch-like segments that construct an inverted tree with a root node, internal nodes, and leaf nodes [28] For this article, a decision tree based on Bonferroni-Holm corrected chi-squared tests was constructed with as target variable the likelihood of refusing the advice to visit the GPC. The independent variables were all patient characteristics, subjective crowding, period of the day, flowchart category and nurse ID. A 10-fold cross validation was used to evaluate the model. The CHAID-analysis was performed using IBM SPSS® version 27.

## Results

### Study population

Of the 6374 patients that presented during intervention weekends, 838 (13.3%) patients were advised to visit the GP and 5456 (86.7%) were advised to be treated at the ED. For 80 patients the advice was unknown. Out of the 838 patients who received the advice to visit the GPC, 599 accepted and were seen by the GP while 183 refused and were treated at the ED [12] The remaining 56 patients left without being seen i.e., were neither seen by a doctor at the ED nor at the GPC. A logistic regression showed that these patients were very similar to those seen by a doctor, in terms of sociodemographic characteristics. The only difference was that those who left, lived nearby significantly more often (OR 2.63, 95%CI: 1.02 to 6.78). In the analysis that follows, these patients are excluded. The 599 (76.5%) patients who accepted the advice were compared with the 183 (23.5%) patients who refused it. For 594 and 169 of these patients, respectively, the invoices were examined.

### Bivariate analysis

The bivariate analysis of the characteristics of non-compliance is presented in Table 1. The results of the patient characteristics show that, while there was no significant age difference, the patient’s sex, socio-economic status, and residence were significantly different between those who refused and those who accepted the advice. Male patients (OR 1.36, 95%CI: 0.98 to 1.90, *p* = 0.07) and patients not living nearby (OR 1.43, 95%CI: 0.98 to 2.08, *p* = 0.07) refused more often. Receiving an increased reimbursement was associated with more refusals (OR 1.37, 95%CI: 0.96 to 1.95, *p* = 0.09). The patient’s flowchart category seemed to have an impact as well (otorhinolaryngology complaints versus unwell adult OR 0.44, 95%CI: 0.22 to 0.89; children versus unwell adult OR 0.51, 95%CI: 0.26 to 1.02, *p* = 0.06). Most patients were assigned urgency category four, while only few were given category five. The urgency categories did not significantly differ between refusers and non-refusers. Almost all patients arrived as a walk-in. Those who arrived by ambulance refused significantly more often (OR 2.84, 95%CI: 1.21 to 6.68). Finally, the timing of the triage also seems associated with the likelihood of refusal. Both subjective crowding at the ED (quiet versus normal OR 0.41, 95%CI: 0.16 to 1.01, *p* = 0.05) and the period of the day (day versus evening OR 0.58, 95%CI: 0.39 to 0.85; night versus evening OR 0.39, 95%CI: 0.39 to 0.66) were significant.

**Table 1:** Bivariate analysis for refusing vs. accepting advice to visit the GPC – characteristics of non-compliance

Determinant		Accept advice (%) (*n* = 599)	Refuse advice (%) (*n* = 183)	p-value	Odds ratio (95% CI)
**Patient characteristics**					
Age	Mean (in years)	30.02	32.78		
	Min – Max	0 – 90	0 – 93		
Age category	0–7	105 (17.5%)	29 (15.9%)	0.88	0.96 (0.54 to 1.71)
	8–24	156 (26.0%)	39 (21.3%)	0.60	0.87 (0.51 to 1.48)
	25–39	156 (26.0%)	53 (29.0%)	0.53	1.18 (0.71 to 1.97)
	40–54	104 (17.4%)	30 (16.4%)		1
	55–74	55 (9.2%)	22 (12.0%)	0.32	1.39 (0.73 to 2.63)
	> 74	23 (3.8%)	10 (5.5%)	0.34	1.51 (0.65 to 3.51)
Sex	Female	318 (53.1%)	83 (45.4%)		1
	Male	281 (46.9%)	100 (54.6%)	0.07	1.36 (0.98 to 1.90)
Increased reimbursement	Yes	207 (37.7%)	72 (45.3%)	0.09	1.37 (0.96 to 1.95)
	No	342 (62.3%)	87 (54.7%)		1
Living nearby	Yes	469 (78.6%)	131 (72.0%)	0.07	1
	No	128 (21.4%)	51 (28.0%)		1.43 (0.98 to 2.08)
**Patient status**					
Flowchart category	Otorhinolaryngo-logy complaints	86 (14.6%)	17 (9.5%)	0.02	0.44 (0.22 to 0.89)
	Children	83 (14.1%)	29 (10.6%)	0.06	0.51 (0.26 to 1.02)
	Others ^a^	122 (20.7%)	10 (16.2%)	0.05	0.53 (0.29 to 0.99)
	Abdominal complaints	76 (12.9%)	21 (11.7%)	0.16	0.62 (0.32 to 1.22)
	Wounds	34 (5.8%)	11 (6.2%)	0.45	0.72 (0.32 to 1.66)
	Limb Problems	75 (12.7%)	31 (17.3%)	0.81	0.93 (0.49 to 1.74)
	Unwell Adult	56 (9.5%)	25 (14.0%)		1
	Back and neck pain	57 (9.7%)	26 (14.5%)	0.95	1.02 (0.53 to 1.98)
Urgency category ^b^	4	578 (96.5%)	171 (93.4%)		1
	5	21 (3.5%)	7 (3.8%)	0.79	1,13 (0.47 to 2.70)
Admission type	Ambulance with or without 112	12 (2.0%)	10 (5.5%)	0.02	2.84 (1.21 to 6.68)
	Walk-in	586 (98.0%)	172 (94.5%)		1
**Timing of the triage**					
Perceived crowdedness	Quiet	41 (17.5%)	6 (8.6%)	0.05	0.41 (0.16 to 1.01)
	Normal	161 (68.5%)	58 (82.9%)		1
	Busy	33 (14.0%)	6 (8.6%)	0.15	0.50 (0.20 to 1.27)
Part of the day	Day	337 (56.3%)	96 (52.5%)	0.005	0.58 (0.39 to 0.85)
	Evening	122 (20.4%)	60 (32.8%)		1
	Night	140 (23.4%)	27 (16.8%)	0.001	0.39 (0.23 to 0.66)

^a^ This category contains chest pain, eye problems, mental complaints, neurological complaints, respiratory complaints, trauma and accidents, urinary or gynaecological complaints, and others. These categories had insufficient observations to be separately included and tested reliably

^b^ Only urgency categories four and five are reported as only five patients in category three and none in categories one and two received the advice to visit the GPC

The bivariate analysis of the patients’ costs is presented in Table 2. First, a distinction was made between the period of the day. Only the total cost during the night and the amount paid by the insurance for patients presenting during the evening or night was not significantly different between those who accepted and those who refused the advice to visit the GPC. For other categories, the cost for the treatment of refusers was significantly higher than that of those who complied. For instance, the average total cost was 76.90 (95%CI: 68.07 to 85.72) euros for refusers, while only 49.86 (95%CI: 47.29 to 52.42) euros for accepters. This is a difference of 27.04 (95%CI:17.86 to 36.23) euros. The overall amount paid for by the patient was on average 20.43 (95%CI: 18.69 to 22.17) euros for refusers, compared to 5.61 (95%CI: 5.12 to 6.10) euros for non-refusers. Furthermore, making the distinction between cost categories indicates that, compared to the GPC, consultation fees at the ED were higher during the day and lower during the evening or night. Other cost components (technical procedures, medication, and non-refundable items) were significantly higher for patients who refused the advice and were treated at the ED (*p* < 0.001). Data on medical imaging ordered by the GPC is unavailable. However, GPs seldomly make use of this. During the second half of the year (July to December) of 2019, imaging was ordered for only 1.3% (95%CI: 1.1% to 1.7%) of the patients who visited the GPC. In contrast, during the trial’s intervention weekends, 45.19% (95%CI: 43.69% to 46.70%) of patients assigned to the ED were charged for medical imaging. It is therefore reasonable to assume that these costs were on average higher for non-compliers.

**Table 2:** Bivariate analysis for refusing vs. accepting advice to visit the GPC– Costs as captured on the patients’ invoice

		Mean in € for those who accept advice (N)	Mean in € for those who refuse advice (N)	p-value two-sided T-test
Total billing	Overall	49.86 (594)	76.90 (169)	< .001
	Day	44.39 (334)	77.49 (90)	< .001
	Evening	51.54 (122)	72.47 (56)	< .001
	Night	61.59 (138)	85.36 (23)	0.154
Billing for patient	Overall	5.61	20.43	< .001
	Day	5.38	21.05	< .001
	Evening	5.21	19.06	< .001
	Night	6.52	21.33	< .001
Billing for insurance	Overall	44.25	56.47	0.006
	Day	39.01	56.44	0.008
	Evening	46.33	53.41	0.180
	Night	55.07	64.03	0.580
**Billing by cost category** ^a^				
Consultation fees	Overall	47.11	46.59	0.625
	Day	42.05	46.93	< .001
	Evening	51.13	47.13	0.096
	Night	55.78	43.94	< .001
Technical procedures	Overall	1.16	12.74	< .001
Medication	Overall	0.12	2.56	< .001
Non-refundable items	Overall	0.05	1.24	< .001
Medical imaging ^b^	Overall		13.76	
**Billing for patient, by cost category** ^a^				
Consultation fees	Overall	5.42	16.53	< .001
	Day	5.23	17.34	< .001
	Evening	5.21	15.09	< .001
	Night	6.06	16.89	< .001
Technical procedures	Overall	0.03	0.87	< .001
Medication	Overall	0.05	1.22	< .001
Non-refundable items	Overall	0.05	1.24	< .001
Medical imaging ^b^	Overall		0.56	
**Billing for insurance, by cost category** ^a^				
Consultation fees	Overall	41.69	30.06	< .001
	Day	36.82	29.59	< .001
	Evening	45.92	32.04	< .001
	Night	49.72	27.05	< .001
Technical procedures	Overall	1.12	11.86	< .001
Medication	Overall	0.06	1.34	< .001
Medical imaging ^b^	Overall		13.19	
**Billing excluding medical imaging and clinical biology**				
Total billing	Overall	48.43	63.13	< .001
Billing for patient	Overall	5.55	19.87	< .001
Billing for insurance	Overall	42.88	43.26	0.872

^a^ The remaining categories were clinical biology and hospital lump sums. These were not included out as only one and zero patients, respectively, had an invoice belonging to these categories

^b^ Data on medical imaging ordered by the GP were not available at the patient level

### CHAID-analysis for accepting vs. refusing advice

**Fig. 1:**
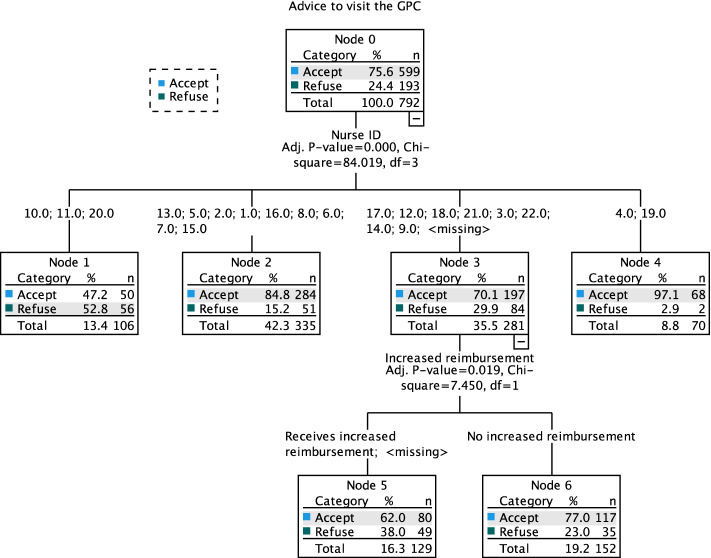
CHAID-analysis for refusing vs. accepting advice to visit the GPC**.** GPC: General practitioner cooperative

**Fig. 2:**
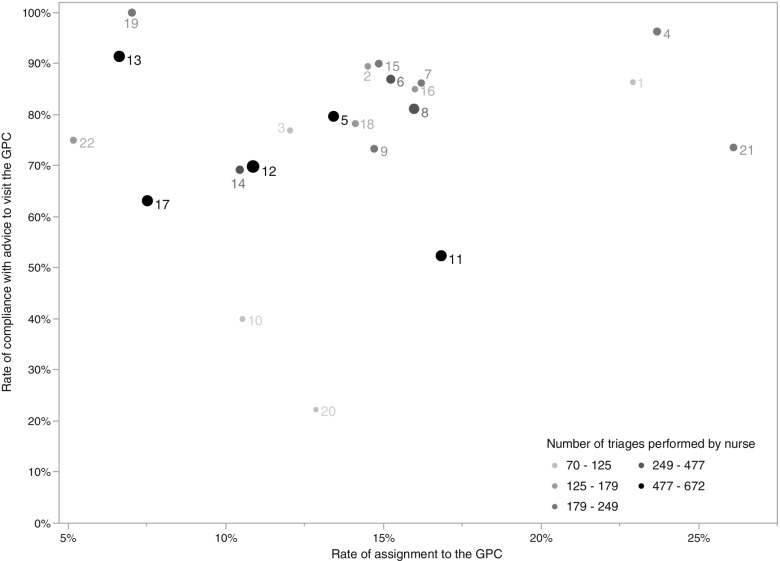
Rate of assignment to the GPC and patients’ rate of compliance, per nurse. GPC: General practitioner cooperative

The CHAID-analysis is presented in Fig. 1 and Table 3. It has seven nodes and a depth of two. The nurse on duty is selected as a first splitting variable (*p* < 0.001). The probability of refusing the advice to visit the GPC was only 2.9% for patients managed by nurses 4 and 19. For nurses 13, 5, 2, 1, 16, 8, 6, 7, and 15 this was 15%. Nurses 10, 11 and 20 had significantly more refusers, namely 52.8%. For patients managed by one of the remaining nurses, the probability of refusing was almost 30%. For this set of patients, economic status was selected as next splitting variable (*p* = 0.02). Patients who received an increased reimbursement had a higher fraction of refusal, namely 38% compared to 23% for those not receiving it. The misclassification risk of the model is 25.5% with a standard error of 1.5%. To illustrate that the importance of the nurse is unrelated to how often nurses advise patients to visit the GPC, the nurses’ assignment and compliance rates are presented in Fig. 2. Two nurses with an outlying low compliance rate (22.2% for nurse 20 and 40% for nurse 10) only triaged 70 and 90 study patients, respectively.

**Table 3:** Statistics of the CHAID-analysis for refusing vs. accepting advice to visit the GPC

Risk			
Method	Estimate	Standard Error	
Resubstitution	.236	.015	
Cross-Validation	.255	.015	
**Classification**			
	Predicted		
Observed	No	Yes	Percent Correct
No	56	137	29.0%
Yes	50	549	91.7%
Overall Percentage	13.4%	86.6%	76.4%

## Discussion

During this trial, 838/6374 (13.1%) patients from the intervention group received the advice to visit the GPC [12] Of these patients, 56 left without being seen. When excluding those patients, 599 (76.5%) patients accepted and were seen by the GP, compared to 183 (23.5%) patients who refused and were treated at the ED. This proportion was mainly influenced by the nurse on duty. This indicates that the nurse delivering the advice to the patient plays a central role in the likelihood of acceptance. This effect is not driven by different assignment rates of nurses. It is not the case that certain nurses have a higher compliance because they advise a smaller share of patients to visit the GPC. One possible explanation for the observed variation in compliance is differences in communication style. During interviews, nurses on duty indicated that communication with patients was key for successful referrals. The practice of referral is not currently embedded in the Belgian habits, hence nurses still had to learn how to best approach patients [29] The remaining variability in refusal was explained by the socio-economic status of the patients. Those receiving increased reimbursement were more prone to refuse the advice to visit the GPC, which was an expected result [18, 19].

Additional significant differences were found. Patients living nearby accepted the advice to visit the GPC more often. It is possible that these patients simply chose the closest available care setting, compared to patients arriving from further, who may have explicitly chosen the ED with the expectation to be treated at the facility. Next, men refused disproportionately often. It is known that men are more likely to visit the ED instead of the GP on call [30] Research on non-compliance with treatment advice is however more ambiguous about the role of patient’s sex [17, 20, 31] The flowchart category proved important as well, indicating that patients may be more worried about certain types of health issues. The perceived nature of the complaint can impact the preference for either the ED or the GPC [8] Moreover, the likelihood of refusing depends on the perceived crowdedness and the part of the day. Compared to a normal crowding level, patients refuse less often when it is quiet at the ED. When it is calmer, the nurse has more time to persuade the patients, resulting in better explanations and arguments [29] Patients are less likely to refuse advice during the day and during the night than during the evening. A possible explanation is that patients are less willing to get into an argument at night or that different types of patients visited during the evening. No previous research found similar results. Finally, it is important to mention that it is possible that additional, unmeasured, differences between compliers and non-compliers exist, such as patients’ primary language or underlying health conditions.

The importance of complying with the advice to visit the GPC is illustrated in the analysis of invoices. On average, the total cost of refusers was 27.04 euros higher than that of accepters. Aggregating this difference over the 196 refusers amounts to an additional 5299.84 euros of possible savings, if non-compliers would have been treated by the GP in the same way as compliers. This is mainly driven by cost differences during the day and the evening. At night, there was no significant difference in total costs. This raises the question whether relocating patients to the GPC is useful during that period, especially since crowding of EDs is less of an issue at night. Although the potential savings are relatively small, they add to the main advantage of relocating to primary care – reduced crowding of EDs – which was the main argument to implement an extended triage. EDs may therefore still consider measures to reduce refusals, if these are not too costly.

For patients, complying with the advice is always financially beneficial. The mean invoice borne by patients was significantly higher for patients who refused and were treated at ED. This is partly a result of the fact that GPC consultations receive high reimbursements by the insurance, such that only about a quarter is paid by the patient. For ED visits, on the other hand, patients bear almost half the cost. The reason is that the ED consultations of patients in the trial were all without referral by a GP, resulting in lower insurance coverage [25] A second driver of the cost difference seems to be that the ED examined patients more extensively, possibly using more expensive resources that are not available at the GPC. It is not known whether these additional resources were necessary or not. A causal effect cannot be isolated as there exists no control group. It is possible that those with more serious and expensive complaints self-select into the group of refusers. It cannot be excluded that refusers correctly identified themselves as in need of ED treatment.

The analysis additionally shows that the cost for the insurance company was not significantly different between refusers and non-refusers during the evening and night. This is due to supplementary consultation fees. During the evening, an additional fee must be paid at the GPC. During the night, additional fees must be paid at both locations, but the amount is higher at the GPC. These fees are entirely born by the insurance and offset the higher ED costs from other cost categories [25].

This secondary analysis of a cluster randomised trial has some shortcomings. First, since the fraction of patients refusing the advice to visit the GPC was not the primary outcome of the TRIAGE-trial, the sample size was not optimal and certain variables were not collected. For instance, the reason why advice was refused and the satisfaction with the received treatment are unknown, as well as some relevant demographic and medical characteristics of the patients. Other variables were simply unobserved or difficult to measure (motivation, distress, etc.). It was therefore not possible to account for all potential confounders. Second, it was not possible to analyse the treatment refusers would have received should they have gone to the GPC. Refusers could only be compared to non-refusers. This lack of control group makes it impossible to state whether observed cost differences were due to the location of care or due to patients’ medical status. This study did not account for the self-selection of refusers and cannot determine whether refusers correctly identified themselves as in need of emergency care. Third, many differences are only significant at the 0.10 level, in part due to the small sample size. Conclusions are therefore explorative rather than definitive. Fourth, patient level data on medical imaging and clinical laboratory tests ordered by the GP were not available. Although such tests are seldomly ordered by GPCs, this may lead to an underestimation of the costs.

Despite these weaknesses, this study offers an important contribution to the existing literature. Previous research focused either on the determinants of low-risk patients visiting the ED [3, 8, 9, 30] or on the determinants of non-compliance to medical treatment [17–21]. This study, however, is the first to gain insights into the determinants of non-compliance with the advice to visit a primary care provider. The analysis was based on the first cluster randomised trial on patient assignment to primary care using the eMTS. It was executed over a long study period and in a real-life setting.

The results allow to propose some targeted intervention. The nurse providing the advice is the most important predictor for non-compliance, indicating the relevance of improving nurse-patient communication. The most appropriate way of conveying a message should be taught to the emergency staff. It is necessary for patients to understand the message. Nurses should make certain that the advice is substantiated and in a clear language, as understanding about treatment decisions is associated with higher compliance [31]. If the concept of GPCs is unknown, it should be explained with a focus on why this type of care is a valuable alternative. Special attention may be required when managing patients receiving increased reimbursement. It may be useful to highlight that the personal invoice is on average four times lower at the GPC. Further research is needed to clarify whether non-compliance is due to poor communication by the nurse or due to patient misinterpretation. This will allow to make more specific recommendations.

## Conclusion

A cluster randomised trial on the assignment of patients from the ED to primary care using the eMTS offered the opportunity for a secondary analysis, studying the determinants of non-compliance with advice to visit the GPC. A bivariate and CHAID-analysis show that the nurse on duty delivering the advice has a crucial role. Interventions to reduce the fraction of refusals should therefore aim to improve nurse-patient communication. The analysis found a considerably higher overall invoice for patients treated at the ED (27.04 euros more expensive on average), illustrating the importance of compliance.

## Data Availability

Given the privacy policy of the iCAREdata database, the authors are not allowed to share the used database. Sharing this database would potentially harm the privacy of the included patients. The Belgian Data Protection Authority does not allow the authors to share the raw data. The authors are able to deliver a selection of variables and the outputs of their statistical software upon reasonable request. Most of the studied data analysed during the current study are available to researchers worldwide after following the application procedures of iCAREdata (see www.icaredata.eu).

## Abbreviations

ED: Emergency department
OOH: Out-of-hours
GPC: General practitioner cooperative
GP: General practitioner
eMTS: Extended Manchester Triage System
CHAID: Chi-square automatic interaction detection
